# Soil microbial strategies for climate mitigation—report from a climate action workshop in Las Vegas, Nevada, February 2024

**DOI:** 10.1093/sumbio/qvae033

**Published:** 2024-12-05

**Authors:** Gwyn A Beattie, Francesca M Cotrufo, Thomas W Crowther, Anna Edlund, Joana Falcão Salles, Jack A Gilbert, Janet K Jansson, Paul R Jensen, Jay T Lennon, Thulani Makhalanyane, Jennifer B H Martiny, Dianne K Newman, Mark Stevenson

**Affiliations:** Department of Plant Pathology, Entomology and Microbiology, Iowa State University, Ames, IA 50011, United States; Department of Soil and Crop Sciences, Colorado State University, Fort Collins, CO 80521, United States; Department of Environmental Systems Science, Institute of Integrative Biology, ETH-Zürich, 8092 Zürich, Switzerland; Oath Inc., La Jolla, CA 92037, United States; Microbial Ecology Cluster, Genomics Research in Ecology and Evolution in Nature (GREEN), Groningen Institute for Evolutionary Life Sciences (GELIFES), University of Groningen, Groningen, The Netherlands; The Netherlands & Department of Microbial Ecology, Centre for Life Sciences, University of Groningen, 9700 Groningen, The Netherlands; Department of Pediatrics, University of California San Diego, La Jolla, CA 92037, United States; Scripps Institution of Oceanography, University of California San Diego, La Jolla, 92037 CA, United States; Center for Microbiome Innovation, University of California San Diego, La Jolla, CA 92037, United States; Biological Sciences Division, Pacific Northwest National Laboratory, Richland, WA 99352, United States; Center for Marine Biotechnology and Biomedicine, Scripps Institution of Oceanography, University of California in San Diego, La Jolla, CA 92037, United States; Department of Biology, Indiana University, Bloomington, IN 47405, United States; Department of Microbiology and the School of Data Science and Computational Thinking, Stellenbosch University, 7602 Stellenbosch, South Africa; Department of Ecology & Evolutionary Biology, University of California, Irvine, CA 92697, United States; Division of Biology and Biological Engineering, Caltech, Pasadena, CA 91125, United States; Division of Geological and Planetary Sciences, Caltech, Pasadena, CA 91125, United States; CUR8, EC2A 2BQ London United Kingdom

**Keywords:** climate, microbiome, soil microbes, plant–soil–microbe interaction, detection of microbial inoculant in soil

## Abstract

Life on Earth faces an existential crisis due to the enduring repercussions of unsustainable human activities since the beginning of the Industrial Revolution. Among the most pressing issues are greenhouse gas emissions, primarily from fossil fuel combustion, unsustainable agricultural practices, as well as the global erosion of the world’s topsoil. While agrochemicals have temporarily increased land productivity, their frequent use has adversely impacted the environment and microbial biodiversity. With half of the global soils already degraded by erosion and a projected 90% at risk by 2050, humanity faces a critical crisis that threatens food production, soil carbon storage, and availability of clean water. In this precarious scenario, microbes and plants may provide promising allies for sustaining life on Earth. Thus, it is crucial for policymakers, scientists, NGOs, and the public to recognize the fundamental importance of the soil microbiome. In February 2024, the workshop “Soil Microbial Strategies for Climate Mitigation” gathered world-leading experts from the most relevant research fields, as well as industry innovators, communicators, artists, and policymakers, to propose soil microbiome-based interventions aimed at enhancing carbon dioxide (CO_2_) drawdown and mitigating soil erosion. The workshop focused on innovative soil microbial inoculant approaches, examining methodologies for measuring soil carbon, enhancing plant health and soil structure, proposing an action plan, and forming collaborative strategies.

Sustainability Statement The Meeting Report, “Soil Microbial Strategies for Climate Mitigation,” presents updates from a workshop that gathered world-leading scientists from diverse fields, as well as industry innovators, communicators, artists, and policymakers. The main objective of the workshop was to identify soil microbiome-based interventions as potential solutions to enhance carbon dioxide drawdown through plant photosynthesis and biomass formation, while also rejuvenating the world’s soils affected by erosion. Soil microbiome-based interventions have the potential to result in improved soil structure and quality, with positive impacts on land and agriculture (United Nation’s Sustainable Development Goals, SDGs 2 and 15), contribute directly to carbon sequestration, Goal 13, and indirectly to other SDGs through local and global effects, including Goal 3 (good health and well-being).

## Introduction

Human activities and their effects on climate change have far-reaching impacts on most life forms on Earth, including microorganisms. Although the detrimental anthropogenic effects on microorganisms are less obvious and less well-characterized, alterations in microbial biodiversity and activity will profoundly influence the resilience of all other living organisms and their capacity to adapt to climate change (Cavicchioli et al. 2019). In particular, the intensification of modern agricultural practices in recent decades, primarily aimed at increasing yields, has largely overlooked environmental repercussions, especially concerning soil health and biodiversity, including macro- and microorganisms (Babin et al. 2021, Averill et al. 2022, Wang et al. 2022).

Soil is the most biodiverse singular habitat, likely home to 59% ± 15% of the species on Earth, ranging from the simplest microorganisms to the most complex organisms, such as mammals (Anthony et al. 2023). Likewise, soils are the basis for producing more than 95% of human food. However, half of the Earth’s soils are degraded to some extent due to erosion, loss of organic carbon and biodiversity, salinization, acidification, compaction, and nutrient imbalance, among other causes due to unsustainable and excessive human activities (FAO & ITPS 2015, Handelsman 2021). Even worse, it is projected that 90% of soils is at risk of degradation by 2050, posing a crisis for human existence. To mitigate a catastrophe and to secure national and economic stability, it is crucial that we develop and implement highly effective soil management protocols that have the highest potential and fastest path to implementation (FAO & ITPS 2015). In this context, microorganisms and plants emerge as the most promising allies to support humanity and help to sustain life in terrestrial areas on Earth (Flemming and Wuertz 2019, Handelsman 2021). Soil microorganisms are fundamental to the existence of all higher terrestrial trophic life forms, playing crucial roles in carbon and nutrient cycling, ecological interactions, the health of animals (including humans) and plants, agriculture, and the global food web (Cotrufo et al. 2015, Cavicchioli et al. 2019). When including ocean habitats, the vast abundance of bacteria and archaea (1.2 × 10^30^ total) (Cavicchioli et al. 2019) and their global biodiversity (10^12^ taxa) (Lennon and Locey 2020) underscore their role in maintaining a healthy global ecosystem (Averill et al. 2022).

Preserving soil microbial diversity should be a mandate for controlling carbon emissions and mitigating climate change. Therefore, as a distinct action toward restoring Earth’s soil health, a 2-day workshop titled “Soil Microbial Strategies for Climate Mitigation” was held in Las Vegas, Nevada, from 14th to 16th February 2024. The Workshop presented a unique collaboration between world-leading scientists from diverse fields, including climate, soil science, microbial ecology, and plant ecology, as well as industry innovators, communicators, artists, and policymakers, who were encouraged to propose solutions. The main objective of the workshop was to identify soil microbiome-based interventions as potential solutions to enhance carbon dioxide drawdown via plant photosynthesis and plant and microbial biomass formation, and root deposits, and microbial necromass formation. Discussions during the workshop focused on the following issues:

- Development and application of innovative soil microbial inoculant techniques, such as creating consortia of natural beneficial soil microorganisms obtained from different geographic regions and enhancing naturally occurring microbial community members *in situ*. These advancements were explored across diverse applications, including precision agriculture, regenerative agriculture, wildland management, and soil restoration efforts targeting varying degrees of soil degradation.
- New and traditional methodologies for measuring soil carbon to accurately determine its flow between different carbon pools, and permanence.

## Participants, structure of the workshop, and talks

**Figure 1.**
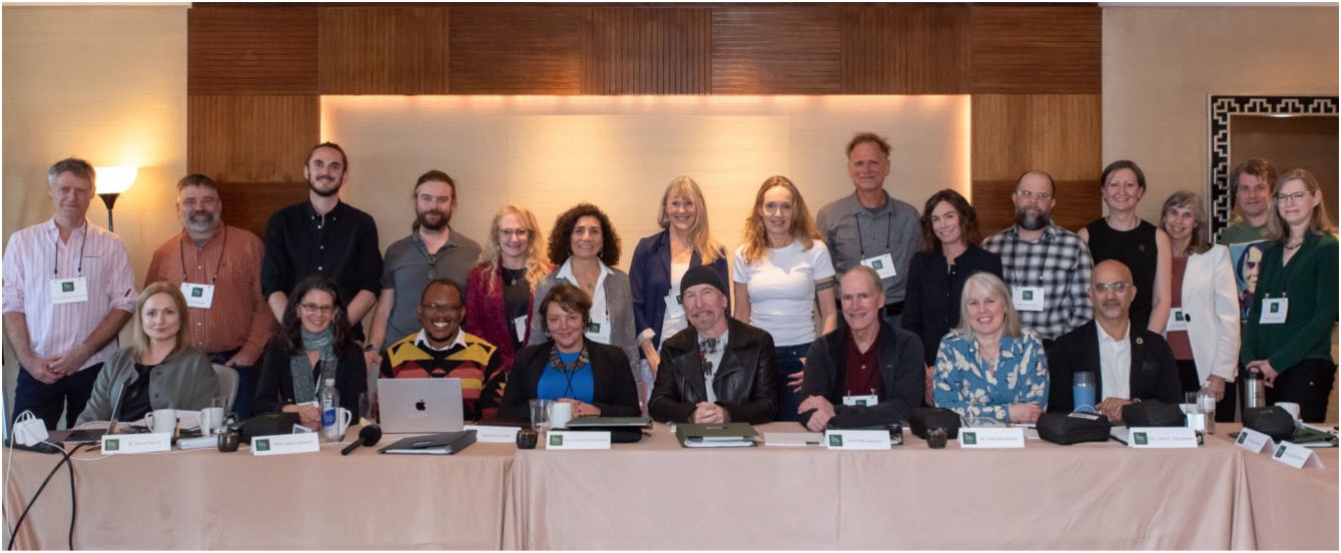
Group photo of workshop participants. Photo with permission from © Oleksandr Kosheliev.

Recognized leaders from the Europe, Africa, and the USA attended the workshop, including (Fig. 1):

**Table:** 

Gwyn A. Beattie	Professor	Iowa State University	United States
Francesca M. Cotrufo	Professor	Colorado State University	United States
Thomas W. Crowther	Professor	ETH Zurich	Switzerland
Anna Edlund	PhD	Oath Inc.	United States
Joana Falcão Salles	Professor	Groningen Institute	The Netherlands
John E. Fernández	Professor	MIT	United States
Jack A. Gilbert	Professor	UC San Diego	United States
Amy Grunden	Professor	North Carolina State University	United States
Jo Handelsman	Professor	University of Wisconsin-Madison	United States
Lucy Harper	PhD	Applied Microbiology International	United Kingdom
Rob Jackson	Professor	Stanford University	United States
Janet K. Jansson	Professor	SAB Chair, Oath Inc.	United States
Paul R. Jensen	Professor	UC San Diego	United States
Jay T. Lennon	Professor	Indiana University	United States
Thulani Makhalanyane	Professor	Stellenbosch University	South Africa
Jennifer B.H. Martiny	Professor	UC Irvine	United States
Dianne K. Newman	Professor	California Institute of Technology	United States
Chris Schadt	Professor	Oak Ridge National Lab	United States
Mark Stevenson	Author, educator	CUR8	United Kingdom
Jan Roelof van der Meer	Professor	University of Lausanne	Switzerland
The Edge	Artist and Special Guest	Rock band U2	Ireland
Gabrielle Walker	PhD	CUR8	United Kingdom

The Workshop was supported by Applied Microbiology International (AMI) and the Microbiome Centers Consortium (MCC). It was arranged around four groups of discussion, each containing experts with diverse backgrounds, who discussed the following issues in an informal style:

- Identification of potential soil microbiome-based solutions to fight against climate crises.
- Identification of any challenges limiting the implementation of such soil microbiome-based interventions to fight against climate crises.
- Identification of solutions for overcoming challenges.

Several inspirational talks were given during the first day of the Workshop addressing the current state-of-the-art of soil microbiome-based interventions, as well as other regenerative alternative practices, such as the use of compost tea and a no-till agricultural management approach.

To kick-off the workshop, a welcome talk was given by Professor Janet K. Jansson, an expert in soil microbial ecology, and one of the workshop organizers. She reminded everyone of the vision of the workshop: *focus on the solutions because we are headed toward an environmental crisis, and we need to do something now*.

Professor Thomas Crowther, ETH Zurich, provided in inspiring talk: “A Global Initiative for Probiotic Efficacy Studies.” He discussed critical aspects of both recreating the biodiversity that has already been lost, and conserving species in already highly biodiverse ecosystems, as well as protecting global habitats, including forests, from a climate change. An emphasis was put on forests and their potential to sequester gigatonnes (Gt) of CO_2_ into plant biomass (225 Gt)—about one-third of the CO_2_ drawdown needed (Mo et al. 2023). Professor Crowther also highlighted the significant impacts of soil microorganisms in forest ecosystems and how they can be employed in restoration efforts (Guerra, Bardgett et al. 2021, Averill et al. 2022). He also proposed that businesses (both small and large) can play a pivotal role to help advancing microbial transplant research and technology developments. He advocated for a global perspective, where soil biogeographies are broken down regionally to identify local microbiome signatures, which then can be enriched and added back to locally degraded soils. Such an inoculant approach would prevent erosion of the natural soil biodiversity in comparison to current approaches where the common practice is to introduce monocultures of foreign microbial inoculants.

**Figure 2.**
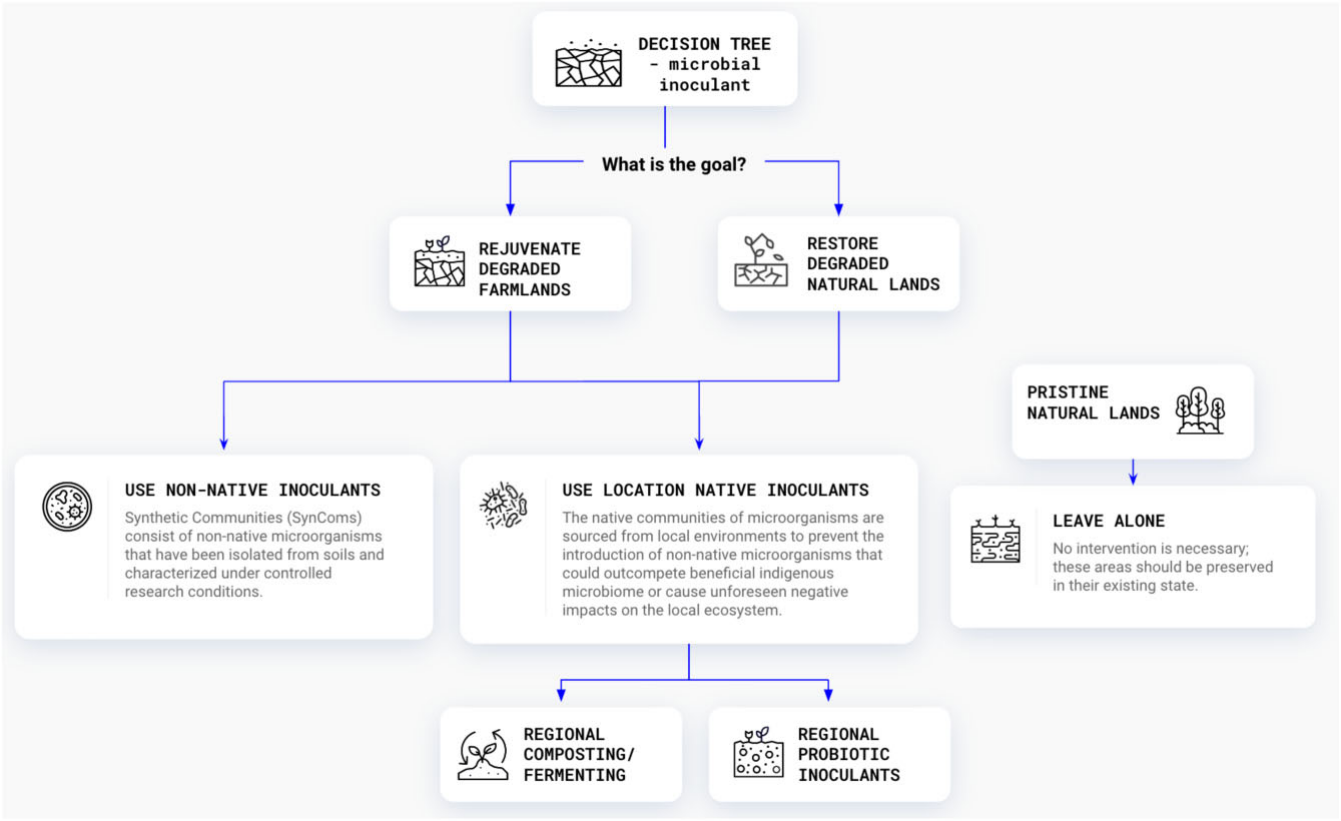
Microbiome-based interventions in relationship to land type. Managed lands, degraded due to human exploitation, can be rejuvenated by applying either Synthetic Communities (SynComs) of non-native, non-GMO microorganisms or native soil microorganisms, in cases where regulatory reasons prohibit the introduction of SynComs. Natural lands, which are non-agricultural lands (or ecosystems) that need intervention, can be restored by applying enrichments of native soil microorganisms. Whereas if the land is not impacted or degraded, the strategy is to leave it alone to allow the natural ecosystem to thrive.

On the second day, action steps toward climate change mitigation using a microbiome-based inoculation approach were formulated. It was noted that these actions are in line with the global interest in ecosystem restoration, highlighted by the “United Nations’ Decade on Ecosystem Restoration” (Aronson et al. 2020). The group concluded that mass restoration projects focusing only on one component of the ecosystem, e.g. just planting trees, can fail because the ecological context (microbiome + soil + plants) is not considered as a cohesive system. Instead, increasing evidence suggests that by taking a more holistic approach and actively restore the soil microbiome, it is possible to significantly enhance the speed, resilience, and success of plants growth in both managed and natural lands (Averill et al. 2022). Experts delineated two main strategies for restoring degraded lands: (i) Rejuvenation of managed (agricultural) lands, (ii), which use distinct beneficial microbiome-based interventions (Fig. 2). Pristine natural lands (lands untouched by humans) were identified as areas that should not be subjected to the introduction of new microbes to preserve their ecological balance and biodiversity.

### Rejuvenate managed land

Managed lands, including agriculture and forestry, cover 50% of the world’s habitable areas. Large-scale monocultures, synthetic fertilizers, pesticides, and reduced genetic variability through selective reproduction, cloning, and genetic modification have increased productivity and efficiency. However, these low-diversity systems are highly susceptible to extreme weather, pathogens, and pests, necessitating heavy agrochemical use (Averill et al. 2022). Moreover, converting natural ecosystems like the Amazon forest or tropical land for agriculture reduces above and belowground species diversity and causes soil microbial homogenization (Guerra, Delgado-Baquerizo et al. 2021, Wang et al. 2022).

To enhance ecosystems and crops sustainably, microbiome-based interventions emerge as potentially effective alternatives. A microbiome is a set of microbial communities or consortia (and their gene repertoires and interactions) associated with an organism (Berg et al. 2020). Both synthetic and native communities of diverse microorganisms can be applied to agricultural lands to boost productivity through low-tech methods such as soil transplants or inoculating co-cultured microbial consortia grown under controlled conditions (Barros-Rodríguez et al. 2021, Averill et al. 2022, Delgado-Baquerizo 2022).

#### Inoculation of synthetic communities of microorganisms

A microbial synthetic community (SynCom) is a term that is commonly used in literature and refers to carefully chosen non-native or native microorganisms that have been isolated as individual strains (not as a consortium) from soils, and characterized, grown, and cultured under controlled laboratory conditions (Jansson et al. 2023). The resulting SynCom-based inoculants can be studied and applied in a controlled manner, which enables development of tailored and stable microbial interactions for enhanced functionality and agricultural benefits, plant health, and soil restoration under normal or stressful conditions. Several studies provide evidence that colonization of beneficial SynComs can promote plant growth and soil health, further supporting their potential to boost crop productivity and soil fertility in sustainable agriculture (Vorholt et al. 2017, de Souza et al. 2020, Shayanthan et al. 2022, Jiang et al. 2023, Martins et al. 2023, Nunes et al. 2024). The global potential of SynComs to enhance agricultural productivity and sustainability is has led to significant public and private investments in microbiome-based products over the past decade (Singh et al. 2020). This growth is driven by the increasing demand for more sustainable agriculture, concerns over pesticide residues, and the pressing need to address climate change, all while ensuring farm productivity increases to sustainably feed a growing population.

#### Inoculation of native and biodiverse consortia of microorganisms

Native microbiome-based interventions may provide benefits such as improved soil health and plant nutrition and defense, carbon capture and erosion control, while maintaining significant microbial diversity (Canfora et al. 2021, Averill et al. 2022). The microorganisms that populate the microbial consortia in principle offer an additional synergy that is not achieved in single-species or exotic microbial inoculations (Babin et al. 2021, Barros-Rodríguez et al. 2021, Shang and Liu 2021, Averill et al. 2022). With multitude of existing antecedents, it is crucial to identify and utilize native, intact, and high-performing microbial communities across various regions worldwide to further develop “regional composting or fermenting” approaches to enhance agricultural productivity. Determining supportive agricultural practices for native microbial consortia will foster the development of effective interventions. Partnerships with scientific organizations, global organizations and companies are crucial to enable responsible microbiome management and scaling, validation and scaling of native microbial introductions.

### Restore natural lands

The inoculation of degraded soils with native soil microorganisms thriving in nearby healthy soils has the potential to restore degraded soils (Averill et al. 2022). In its simplest form, transplantation consists of moving soil and its associated microbial communities from one location to another (Aguilar-Paredes et al. 2020). A meta-analysis of 284 studies further showed an average 64% increase in vegetation biomass with microbiome transplants, with effects reaching as high as 700% (Crawford et al. 2019). Transplant methodologies have several advantages, such as being a low-tech method, having proven track of results far outweighing those methods inoculating commercially available soil microbial mixtures, and preventing the introduction of non-native microorganisms that could outcompete beneficial native microorganisms, or cause unforeseen negative impacts on the local ecosystem (Averill et al. 2022). While promising, soil transplants pose challenges because they are not universally effective and depending on the ecological context. Also, as soil resources are finite, there is a risk of damaging donor sites and damaging the habitat. Thus, developing non-destructive methods to introduce native microbial consortia via transplantation would be highly beneficial.

## Microbiome-based interventions to facilitate CO_2_ drawdown and soil carbon stabilization

The expert groups also discussed the main challenges concerning CO_2_ capture methods integrity using soil microbiome-based interventions, and the following concerns and questions were raised:

- Is there enough evidence that selected soil microorganisms have proven benefits to enable their widespread application?
- Do microbiome-based interventions provide the necessary durability of carbon in the soil, which would justify reliable investments in carbon credits?
- To what extent does soil type, geography, climate, and biodiversity any effect on CO_2_ sequestration rates by plants and associated soil microbes?
- Can plants be bred/treated/stimulated to attain deeper roots and thereby improve soil carbon permanence?
- Can plant-microbiome interactions be optimized or even maximized to induce plant immunity and plant growth at the same time as they help to sequester and store soil C?

## Conclusion

The “Soil Microbial Strategies for Climate Mitigation” workshop held in Las Vegas in February 2024 emphasized the opportunity to enlist soil microorganisms to help mitigate negative consequences of climate change. The attending experts reached a consensus on five overarching goals for research and policy aimed at the implementation of soil microbiome-based interventions (Table 1). They highlighted the significant impact of human activities on microbial biodiversity and the importance of soil health for climate resilience. Key discussions focused on developing and rigorously evaluating innovative soil microbiome-based interventions that can enhance soil carbon sequestration to reduce atmospheric CO_2_ levels. The workshop called for establishing soil health metrics, incentivizing best practices, and recruiting soil advocates to ensure sustainable food production and environmental conservation. Actionable goals included fostering collaborative studies that involve local farmers, landowners, academia, and businesses to advance this field; hosting future workshops to further inspire the development of translational testing and implementations of soil inoculants; publishing relevant research; and contributing to the formulation of governmental policies and climate goals.

**Table 1.** Goals for research and policy for use of the soil microbiome to mitigate climate change.

Goal	Action
Obtain consensus on the facts: Address skepticism, risks, and biases surrounding the plausibility of soil regeneration using scientifically peer-reviewed and published data.	Publish statistically valid scientific publications based on laboratory, greenhouse, and field trial data. Educate and train relevant stakeholders in an unbiased manner.
Obtain metrics of soil health and how to achieve it.	Invest in baseline soil research to define soil health metrics (biological and chemical variables), observe them in real-time (apply new technology: inexpensive, scalable sensors), and understand why things are the way they are.
Incentivize adoption of best practices.	Develop programs and incentives that help farmers transition to soil-protective practices. Foster microbial production of refractory carbon to produce carbon-rich soils: use of cover crops, and cultivation of deep-rooted mixed perennials on median strips. Develop microbial interventions and protocols to restore soil health.
Reduce land use for animal farming.	Integrate microbial interventions with regenerative agricultural practices to produce diverse and healthy crops for food.
Recruit soil advocates and ambassadors	Provide knowledge of soils and microbiomes to all, and mobilize action to protect and restore soil health, ensuring the sustainability of food production, environmental conservation, and climate resilience.

Recognizing and harnessing microbial power holds great promise for creating a resilient and resource-efficient agricultural future capable of addressing the escalating threats of climate change. Soil microorganisms, through biofertilizers and biopesticides, have the potential to revolutionize traditional and precision agriculture, ensuring global food security while mitigating climate impacts. To realize these benefits, fundamental knowledge gaps must be addressed, including the efficacy of inoculated microorganisms under diverse field conditions, their interaction with native microbiota and plant species, and the development of new culture and production technologies. Understanding these aspects will enable the successful commercialization of microbiome-based inoculants and their long-term persistence in soil, paving the way for sustainable agricultural practices and climate mitigation.

## Data Availability

No other data source.
